# A Look at CMAJ: A Misty Image Indeed

**DOI:** 10.4103/0973-1229.27603

**Published:** 2006

We start a new series with this issue.

The Looking Glass will depict how others look at medical practice, its practitioners, mental health workers and philosophers. It will also reflect on happenings in the world of Medicine and Science.

We start with Medical Journals. The *CMAJ…*

The date: Feb 20, 2006. Medical publishing was rocked by the sudden dismissal of the Editor In Chief of the *CMAJ* (Canadian Medical Association Journal), John Hoey, and a Senior Deputy Editor Anne Mary Todkill. The Editor appointed in the interim, Stephen Choe and another Deputy Editor Sally Murray, also resigned in a week's time, on 28 Feb to be precise. Along with them also went Jerome Kassirer, a former *CMAJ* editorial board member and a former editor of the *New England Journal of Medicine (NEJM),* appointed to frame regulations or governance plans about editorial independence.

All these are well-respected professionals in the field of biomedical publication and there has been a huge outcry, both in the medical as well as popular press, at their abrupt sacking.

If this were not enough, fifteen out of the nineteen members of the editorial board of the *CAMJ* also resigned, precipitating a grave crisis. In the meanwhile, the CMA, in an attempt at damage control, appointed an interim Editor, Noni Macdonald, along with an Editor Emeritus, Bruce Squires, who has been an earlier editor of *CMAJ*, and a founder member of WAME. A retired Chief Justice of the Canadian Supreme Court has been appointed to chalk out a Governance Plan for Editorial independence. Changes are also envisaged in the JOC (Journal Oversight Committee). The CMA President is exhorting everyone concerned to move on, and has assured a policy of editorial independence. The atmosphere, at the time of writing this, is combative and smoldering, and a distinct unease prevails.

## Long Standing Feud

There has been a long-standing feud between the CMA, the organisation that owns and tries to control *CMAJ*, and the Editor of *CMAJ* over editorial independence. John Hoey has been asking for greater editorial independence since the journal, although belonging to the CMA, according to him, actually belongs to the whole world of medicine and science, and is, really speaking, accountable mainly to them.

John Hoey, in nearly a decade at the helm (he was hired in August 1996), has brought *CMAJ* from a modest journal to one whose impact factor is today fifth in the world of general medical journals, only less than that of the *NEJM* (38.6), JAMA (24.8), *Lancet* (21.7) and *BMJ* (7.0). He brought it from an impact factor of 1.6 in 1997 to an impressive 5.9 in 2004. (It has since gone to 7.4 in 2005. See ‘About *CMAJ*’ at *: http://www.cmaj.ca/misc/about.shtml).*

What has happened with the *CMAJ* closely approximates what happened with *JAMA* in 1999 when George Lundberg, the editor for 17 years at *JAMA* (Jan 1982 onwards), and who brought it to scientific respectability, was summarily sacked by the executive vice-president of the American Medical Association (AMA), E Ratcliffe Anderson, Jr., during the Bill Clinton oral sex episode. He was sacked because he fast tracked an article on college students′ perception whether oral sex constituted sex (Sanders and Reinisch, 1999). A predominant section, 59%, felt it did not. Now this coincided with the Bill Clinton-Monica Lewinsky episode, for which the Republicans wanted to impeach the President. And the publication of such a report in a prestigious Journal like the *JAMA* may have acted to blunt the opposition. The AMA, which owns the *JAMA*, predominantly supported the Republicans. Such a fast track publication by the powerful Editor was thought to be a political move by the Association office bearers. Although, mind you, it was peer reviewed and accepted for publication in a proper manner. But the fact that it was fast tracked to coincide with this episode was enough to precipitate the sacking. And no amount of outcry that the Journal fast tracked not for any other reason but that it was topical convinced the Association to change its stance. For further reading connected to this, please refer to Hoey, Caplan, Elmslie *et al* (1999); Smith (1999a, 1999b); Van Der Weyden (1999); Kassirer (1999a); and Horton (1999).

The same year, Jerome Kassirer, Editor of *NEJM*, was sacked because he insisted the Massachusetts Medical Society, which owns the journal, not use the name of *NEJM* for ancillary products as it may increase the credibility of the latter but ran the danger of reducing the credentials of the former, carefully developed and crafted by Editors and their Boards by decades of hard work. Well, the argument too did not cut much ice with the association wanting to cash in on the *NEJM* name, and he was fired too. Marcia Angell, who came as interim Editor-in -Chief, too did not last long at the helm. For those who need to be updated, she went on to write the reasonably well read, *The Truth About Drug Companies: How They Deceive Us And What To Do About It* (Angell, 2004) after that, as she appeared fed up with the way pharma tried to manipulate medicine, and medical journals too. Kassirer too, interestingly, went on to write his own book on a similar theme: *On the Take: How Medicine's Complicity with Big Business Can Endanger Your Health* (Kassirer, 2004). For details of the Kassirer episode at *NEJM,* see Parmley (2000), Hoey (1999), Kassirer (1999b) and Angell (1999).

The salutary effect of sacking the editor of *JAMA* was establishment of an Editorial Governance Plan (Signatories of the Editorial Governance Plan 1999; DeAngelis and Maves 2004), as well as discussion on editorial governance and independence by concerned academicians and editors (Davies and Rennie, 1999). The *JAMA* since then has had a relatively smooth sailing.

## What Sparked It All

The *CMAJ* episode was sparked off by two separate but related incidents (although, to be fair, the CMA and the CMA Media Inc President deny any such links).

One was in September 2005, when a Plan B morning-after pill (levonorgestrel), a pill for contraception to be used by females, was investigated in the *CMAJ*. (It is not only a morning after pill. Well it is in a way, for supposed to be taken the morning after, but effective if taken up to even 72 hours later). The pharmacists had a big stake in the product. It was changed from a prescription to a non-prescription emergency contraceptive drug, but with an important rider. The pill was costly enough, but the pharmacist was supposed to charge almost an equal amount for counselling about the appropriateness of the drug. Moreover, they were supposed to collect personal details, including sexual history, during this counselling. The *CMAJ* probed this by asking a few ladies whether they approved of this, which obviously they did not (Eggertson and Sibbald, 2005). The Canadian Pharmacists Association objected, saying a medical journal had no business to do investigative journalism. The CMA, the controlling body, concurred with the pharmacists for obvious reasons. Also, they probably wanted a stick to beat an uncompromising editor with. The article was published with alterations suggested by the CMA. But the Editor wrote an editorial on 3 January 2006 (early release 12 December 2005), denouncing the act, accusing the publisher of editorial interference since they removed a sidebar to the journal's news article which suggested that pharmacists were infringing on women's privacy rights by demanding and registering personal information on the Plan B contraceptive (*CMAJ*, 2006):

We have a transgression to report. While the Dec. 6, 2005, issue was in preparation, the editorial independence of the journal was compromised when a CMA executive objected strenuously to a news article we were preparing on behind-the-counter access to emergency levonorgestrel (Plan B). The objection was made in response to a complaint from the Canadian Pharmacists Association, who had learned about the article when they were interviewed by our reporters. The CMA's objection was conveyed to CMAJ's editors, and to our publisher, who subsequently instructed us to withhold the article.

The stated objection was to our reporting method; as one component of the story, we had asked 13 women from across Canada to attempt to purchase Plan B from a pharmacy in their community and then tell us what the experience was like. The CMA questioned the propriety of our investigation and the boundary between news reporting and scientific research. Our story was not scientific research, however, but legitimate journalism (CMAJ, 2006).

The editorial further said:

We felt that we had a choice between pulling the entire story, or getting most of it out by publishing a negotiated revision. We opted for the latter: what our readers saw omitted the results of our informal survey. This transpired without the story having been read by those who were raising the objection.

Our objective in making this incident public is to set in motion a process to ensure the future editorial independence of the journal. Readers expect CMAJ editors to select content without interference, and authors expect their work to be judged without regard to the interests of any third party. Readers and news media who rely on our reporting need to know that our journalists are not subject to censure (CMAJ, 2006).

There has been a history of confrontation between the Editor of *CMAJ* and the CMA. On Sept 17 2002, the journal had carried an editorial criticising Bill 114, a proposed Quebec legislation that was meant to force family physicians to work on emergency duty during shortage of staff (*CMAJ*, 2002). This had lead to an altercation between the then CMA President Dana Hanson who called it, on October 29th, 2002, ‘seriously flawed’ and said further that the ‘conclusion that physicians have betrayed a trust which we all hold at the very heart of medicine is repugnant’ (Hanson, 2002). On November 26th 2002, the *CMAJ* Editorial board called it a ‘clear and present danger’, in a letter in the journal, and said ensuring editorial independence was critical to its progress (Armstrong *et al*, 2002). They said further:

Whether or not one agrees with the opinions stated in the Sept. 17 editorial is not the fundamental issue here: rather, it is the right to articulate such an opinion without concern forretribution by an organization or corporation that holds ownership or operating responsibility for the journal (Armstrong et al, 2002)

In December 2002, a Journals Oversight Committee to ensure smooth functioning between the *CMAJ* and CMA was formed and had its first meeting. The love-hate relationship between a prosperous Journal wanting greater editorial independence and a wary Association trying to control and clip its wings continued till the Plan B episode, in which a powerful lobby like the pharmacists′ interests were involved.

The other precipitating factor, the final straw in a way, was critical remarks on the coming to power of the present Health Minister of Canada, Tony Clement. He is known to favour privatization and corporatisation of the health sector by introducing private provision within the public sector. The CMA supports this move. It will obviously benefit both CMA members and the pharmaceutical industry. Both important constituencies, and both would want, and support, such a move. Kassirer *et al* (2006) give the lowdown on this rather murky state of affairs:

As this report on the Plan B commentary was being finalized, we became aware through a communication of the Canadian Health Coalition that another news story published electronically on Feb. 7, 2006, was subsequently removed from the CMAJ Web site (online Appendix 1, www.cmaj.ca/cgi/content/full/174/7/945). The article was a report on the appointment of the federal Minister of Health by the new Conservative government. It pointed out the health minister's favourable stance toward privatization of health care delivery during his tenure as the Minister of Health for Ontario. On Feb. 22, 2006, a different report on the federal Minister of Health appeared in the original's place (online Appendix 2, www.cmaj.ca/cgi/content/full/174/7/945). Though the revised article contains some of the same phraseology as the original, it is more supportive and less critical of the health minister and seems more beneficial to the CMA. We pose the question as to whether the extensive revision of this article is another instance in which the political interests of the CMA exerted an influence on CMAJ publishing decisions. Some days before the firing of the editor in chief and senior deputy editor, the JOC was informed about a disagreement concerning the original Tony Clement article, but the JOC turned down a request for an emergency meeting. The editors are not willing to comment on how the changes came about; the publisher has also declined comment.

Under such circumstances, to continue to have an upright editor at the helm of an important opinion maker like the *CMAJ* would have been risky. Already relations were strained between the CMA and *CMAJ* Editor. To expect the latter to toe the CMA line was improbable. Hence, the CMA, under cover of its associate company CMA Holdings (recently changed to CMA Media Inc) used the services of a newly appointed President, Graham Morris, to do the act. The salvo was fired in a three-paragraph termination by the last named gentleman. (Well, English is a civilized language):

The Canadian Medical Association Journal (CMAJ) announced that Dr. John Hoey would be leaving his post as Editor-in-Chief today.

During the ten years that Dr. Hoey has been Editor-in-Chief of CMAJ, he has broadened the scope of the publication and raised its international reputation as a respected peer-reviewed scientific journal.

The CMAJ's mission in serving its 69,000 readers is to provide accurate and up-to-date scientific and clinical information on the promotion of health and the treatment of disease (Media Advisory, 2006).

So, if the first reason was economic, the second was political. And the two scourges of modern medicine, economics and politics, managed to do in the credibility of a respectable Journal. While we know the economic-political compulsions of Associations and its office bearers, that they could not rise above their petty considerations in the larger interest of science and research is a sorry tale to recount.

The outcry at such a summary dismissal has been unanimous. Editorials by reputed journals like the *Lancet* (2006), the *BMJ* (Godlee, 2006), *MJA* (Van Der Wayden, 2006), articles in the *NEJM* (Shuchman and Redelmeier 2006; Hoey 2006), as also the ICMJE (2006) and WAME (2006), and even the lay press, have all decried the act. One of us has written rather copiously on the topic in the WAME List serve and elsewhere (Singh 2006a–e). The editors concerned have not spoken earlier about the action because they are purportedly bound by a confidentiality clause, but Hoey has since written an article discussing editorial independence (Hoey 2006) in the wake of the *CMAJ* crisis; while the CMA president has promised full editorial independence to the interim editorial board and editors, and urged all concerned to move ahead (Sullivan, 2006). At the time of writing, an interim editor, Noni Macdonald, and Editor Emeritus, Bruce Squires, are handling matters since March 7, 2006 (Shibbald, 2006).

## A Private Matter Between Employer And Employee?

Some of you may feel this is not an issue for us to discuss at all. It is only a private matter between an employer and an employee. We beg to differ. This will be the argument of any employer who holds the cards, and would want to regulate the game. For long have we believed that he can.

It is not *only* a private matter between an employer and an employee. The product of this employer-employee interaction is scientific knowledge and research advancement, which are of great importance to society. Hence, let us realise that it is, really speaking, a matter of scientific concern, biomedical advance, ethical conduct, and editorial independence. For all of which we toil day in and day out. Our unequivocal stand will have far reaching ramifications if we do not allow our minds to be paralysed by analysis of subsidiary concerns.

The *Mens Sana Monographs* wishes to record, in no uncertain terms, that this is a sorry and murky state of affairs which needs to be speedily and effectively remedied.

It demands an action plan:

## The Action Plan

What actions need be taken? We think they are basically five, based on the principles of peaceful non-cooperation:

1. The entire board of present editors should resign in protest against the firing. And not rejoin till the sacked and resigned editors are reinstated. They must be absorbed in other journals if it comes to that, so their positions are secure.
2. The WAME and ICMJE should categorically support the reinstatement of the fired editors. No mincing words, just clear-cut statements of strong protest and expectation of appropriate action. And follow up by supporting the agitating board members, as well as the fired editors, with legal counsel and financial help for the protracted battle to follow.
3. An agitation for reinstatement of the fired editors by members of CMA, lead by members whose credentials are aboveboard and have no political/ personal agenda of their own to fulfill.
4. The research community whose articles have been accepted by *CMAJ* must ask for withdrawal of the accepted articles, and researchers should decide not to submit their research work to a journal which does not respect editorial independence. Similarly, reviewers should refuse to review papers submitted.
5. Questions must be raised in the Canadian parliament, and the CMA asked to answer on the floor of the house why it took such an action

## The Inevitable Conclusions

As the dust settles on this episode, which it inevitably will, (although from all portends, the feisty members of the *CMAJ* Editorial Board are far from relenting at the present time), some things become clear.

1. Journals and Editors, for all their uprightness and scientific merit, since they are under the thumb of Associations and its office bearers, are always walking a tight rope. Whenever they appear inconvenient to the latter beyond a point, they will always be summarily dismissed.
2. The outcry, loud and impassioned, will as surely abate, because it lacks the teeth to convert its anger into collective action.
3. The Editors will lose any battle in this fight, for the odds are stacked against them. This in spite of the fact that they are on the right side.
4. History will continue to repeat itself.

This analysis can of course be disproved by action that a determined editorial board and other supporters take. And we much wish it happened. Just once. For a strong arbitrary action by an employer needs an equally strong spirited response from the editors/readers/members of CMA/researchers.

Once one editor of integrity is reinstated. Only once. No employer, or Association President, or commercial interest, will ever dare touch, or manipulate, legitimate editorial freedom.

Every time we only make noises and accept, every time editors will be upturned when they become inconvenient. And nothing substantial will change, except for the filling up of journal and discussion pages with high-sounding principles.

Well, if we want to change History, or rewrite its course, we need the steel to go all the way.

This is not the time to resign to fate, or get cynical about the lack of morals or principals. That only justifies our inaction, and emboldens the opposition.

The fate of the earlier editors of *JAMA* and *NEJM* need not be repeated in *CMAJ*, or for that matter anywhere else. Those who are keeping silent and are upright should realise that if they do not join the fight today, the axe will fall on them tomorrow. So they either toe the establishment line, or fight. It is suicidal to have the ostrich attitude.

It is of course premature to depict where this fight will end. But, in any case, we can look into some long-term effects of such confrontations.

## The Long Term Sequelae

1. Every fight for editorial independence by upright editors, even when they are sacked, is eventually for the good. For, in the wake of the outcry, managements have to spell out with greater clarity where and when will they intercede. This itself is a significant step. For example, JOC (Journal Oversight Committees) were set up in the wake of the *JAMA* and *NEJM* affairs. Even the *CAMJ* episode has seen the JOC play a role, although not that worthy a role as many would have wished. But then, probably that is the fate of all middlemen. Liable to be lashed from both sides. Ultimately, with every such action, although a battle in the form of an editor sacked is lost, the war for editorial independence is being won. It may sound paradoxical, but it is surprisingly true.
2. Associations, which own journals, will have to decide to give up ownership rights over journals. This must come sooner or later, and the sooner the better. The financial and other logistics have to be worked out, of course. But most prestigious journals manage to earn revenues for themselves too, so it is not as formidable a task as may first appear.
3. Those Journals that still depend on Associations may work out a system like the *BMJ* where the Editor is appointed by the Council of the BMA and, if at all, would have to be fired by it. He/she is on par with the Secretary of the Association (here the BMA), something equivalent to the Executive Vice-President of the AMA for example, and reports directly to a Council which is the servant of the Annual Representative Meeting, and also if needed to the Annual Meeting of the BMA. Not to any individual who can hire or fire him. The major difference between BMA and many other medical associations, including those like the AMA, as also the Australian and the Canadian, is that the Editor does not report to the Secretary. In fact both are chief joint executives. This has ensured the reduced coming and going of editors in the *BMJ* for example, with hardly such problems as the *CMAJ* faces today, or the *NEJM* and *JAMA* faced not so long back. It proudly claims it has had only six editors in a century, certainly an enviable record, and worthy of emulation (Smith, 1999b). The Editorial Governance Plan at *JAMA* is another worthy claimant (Signatories of the Editorial Governance Plan 1999; DeAngelis and Maves 2004).
4. Other solutions are when the Journal publishes the Presidential Address or Orations/Award Papers of a Society/Association, and has a regular column for Events/Announcements of the Society/Association, but with regard to everything else, it is absolutely independent. Such a step is essential if a Journal has to earn respectability as a biomedical publication and not become only an Association mouthpiece or Bulletin.
5. A Society/Association should know that a Journal's reputation, beyond a certain limit, is independent of its originator. It must reach the broader constituency of the world of biomedical science and researchers spread all over. It must allow for, even encourage such flowering, for in so doing the Association truly appreciates the potential of its worthy progeny. It is very much like a father and a son. The son needs the father in the initial stages. But when he outgrows that need, it is suicidal for the father to continue to control, or try and milk the son. That can only lead to bitterness and rancour. What western society has forsaken in its family structure by giving up on the joint family, unfortunately, it is still clinging to here, creating untold hardships in the bargain.
6. Editors should not, reading the writing on the wall, become handmaidens of the establishment. They may survive, even prosper, that way, but will never leave their impress ‘on the sands of time’. Their role, fundamentally, is to further scientific advancement, irrespective of the compromises circumstances force on them. That does not sanction gladiator roles, nor does it mean every editor must perennially fight against an establishment. It just means the Editor, and the Journal he heads, by their very nature, have to be in pursuit of scientific evidence and patient welfare. That may occasionally bring them in conflict with powerful economic, political and bureaucratic forces, including Association office bearers. But that they must accept as a professional hazard, and plod on nevertheless.
7. No consideration or power that tries to over ride *scientific evidence* and *patient welfare*, the twin pillars of biomedicine, howsoever high and mighty, can become a predominant concern for an Editor, or Journal. Ever. That is the bottom line, and the very raison d′ etre for their existence.

If ever a lesson has to be reiterated, this episode and its unfolding aftermath should italicise it.

Hopefully.

**Ajai R. Singh**

**Shakuntala A. Singh**

Editors, *Mens Sana Monographs*
